# Identification of the Representative Primary Biliary Cholangitis Cohort in Taiwan: A Comparison of Four Nationwide Cohorts

**DOI:** 10.3390/jcm10112226

**Published:** 2021-05-21

**Authors:** Cheng-Jen Chen, Jur-Shan Cheng, Haw-En Wang, Chun-Wen Huang, Jing-Hong Hu, Wei-Ting Chen, Ming-Yu Chang, Hsin-Ping Ku, Cheng-Yu Lin, Rong-Nan Chien, Ming-Ling Chang

**Affiliations:** 1 Division of Hepatology, Department of Gastroenterology and Hepatology, Chang Gung Memorial Hospital, Taoyuan 333423, Taiwan; k85731@cgmh.org.tw (C.-J.C.); krosiswang@hotmail.com (H.-E.W.); mp1001@cgmh.org.tw (C.-W.H.); weiting1972@gmail.com (W.-T.C.); find94132@yahoo.com (H.-P.K.); 8805035@cloud.cgmh.org.tw (C.-Y.L.); ronald@adm.cgmh.org.tw (R.-N.C.); 2 Department of Medicine, College of Medicine, Chang Gung University, Taoyuan 333323, Taiwan; 3 Clinical Informatics and Medical Statistics Research Center, College of Medicine, Chang Gung University, Taoyuan 333323, Taiwan; jscheng@mail.cgu.edu.tw; 4 Department of Emergency Medicine, Chang Gung Memorial Hospital, Keelung 204, Taiwan; 5 Division of Gastroenterology and Hepatology, Department of Internal Medicine, Chang Gung Memorial Hospital, Yunlin 638, Taiwan; a3237184@gmail.com; 6 Division of Pediatric Neurologic Medicine, Chang Gung Children’s Hospital, Taoyuan 333423, Taiwan; p123073@gmail.com; 7 Division of Pediatrics, Chang Gung Memorial Hospital, Keelung 20401, Taiwan; 8 Liver Research Unit, Department of Gastroenterology and Hepatology, Chang Gung Memorial Hospital, No. 5, Fu Hsing Street, Kuei Shan, Taoyuan 333423, Taiwan

**Keywords:** PBC, prevalence, incidence, outcome, ICD-9

## Abstract

Background/Purpose: The rates and outcomes of primary biliary cholangitis (PBC) in Taiwan remain unclear. Methods: A nationwide population-based cohort study (Taiwan National Health Insurance Research Database, 2002–2015) was conducted. Data from four PBC cohorts with various definitions were compared (cohort 1 (C1): ICD-9-CM (571.6); C2: alkaline phosphatase (Alk-P) and antimitochondrial antibody (AMA) measurements; C3: Alk-p and AMA measurements and ursodeoxycholic acid (UDCA) treatment; C4: ICD-9-CM (571.6), Alk-p and AMA measurements and UDCA treatment). Results: The average prevalence rate ranged from 9.419/10^5^ (C4) to 307.658/10^5^ (C2), and the female-to-male ratio ranged from 1.192 (C1) to 3.66 (C4). Prevalence rates increased over time in all cohorts. The average incidence rates ranged from 1.456/10^5^ (C4) to 66.386/10^5^ (C2). Incidence rates decreased over time in C1 (−9.09%, *p* < 0.0001) and C4 (−6.68%, *p* < 0.0001) and remained steady in the others. C4 had the lowest prevalence and incidence rates and highest female-to-male ratio. Cirrhosis rates ranged from 7.21% (C2) to 39.34% (C4), hepatoma rates ranged from 2.77%(C2) to 6.66%(C1), liver transplantation (LT) rates ranged from 1.07% (C2) to 6.77% (C4), and mortality rates ranged from 18.24% (C2) to 47.36% (C1). C4 had the highest LT (6.77%), osteoporosis (13.87%) and dyslipidemia rates (17.21%). Conclusions: Based on the reported ranges of reasonable rates, female predominance and characteristic outcomes, C4 was the most representative Taiwanese PBC cohort, with average prevalence and incidence rates of 9.419/10^5^ and 1.456/10^5^, respectively, and a female-to-male ratio of 3.66. In a 14-year period, cirrhosis, hepatoma, LT, and mortality were noted in 39.34%, 5.52%, 6.77%, and 34.22% of C4 patients, respectively.

## 1. Introduction

Primary biliary cholangitis (PBC), formerly called primary biliary cirrhosis [1], is an idiopathic chronic autoimmune liver disease that predominantly affects middle-aged women [2]. Without treatment, most PBC patients eventually develop hepatic fibrosis and may require liver transplantation during the late disease stage [3]. Ursodeoxycholic acid (UDCA) and obeticholic acid (OCA) are currently the two licensed therapies for PBC [4]. However, some patients treated with UDCA or OCA do not exhibit adequate responses, as only 60–70% of PBC patients treated with UDCA exhibit complete responses [5], and up to 60% of UDCA suboptimal responders do not have a therapeutic response to OCA [6]. The early detection and treatment of PBC is therefore important to prevent complications [7].

Most of the information on PBC has been derived from studies conducted in Europe [4] or the USA [2]. Nationwide epidemiological data on PBC are lacking in Taiwan, an Asian country where OCA is still not available for the treatment of PBC. Several population-based studies have defined PBC based on administrative data on the International Classification of Diseases, Ninth Revision, Clinical Modification (ICD-9-CM:571.6), or ICD-10-CM, codes and used those data to determine the prevalence or incidence rates of PBC in various countries [8,9,10,11,12,13]. However, in addition to PBC, the ICD-9-CM code 571.6 represents secondary biliary cirrhosis, which is not uncommon in Taiwan, where hepatolithiasis is prevalent [14]. Therefore, the number of true PBC cases might be less than the reported number, and biases may arise from differences in coding practices across different clusters [10]. While the ICD-10-CM code K74.3 is specific for PBC, it was not used in Taiwan until 2016; thus, the precise annual incidence rates for PBC in Taiwan cannot be estimated based on this ICD-10-CM code as the washout time for surveying incidence rates, which should be more than 4 years, has not yet been reached [15].

Accordingly, we conducted a retrospective study with data from the Taiwan National Health Insurance Research Database (TNHIRD). Four PBC cohorts defined with various combinations of the ICD-9-CM code, biochemistry measurements and/or UDCA administration were assessed. The associated prevalence and incidence rates and outcomes were compared among the four PBC cohorts to identify the most accurately representative PBC cohort in Taiwan.

## 2. Methods

### 2.1. TNHIRD Samples and Measurements

Given that positivity for the antimitochondrial antibody (AMA) with elevated alkaline phosphatase (Alk-p) levels are used to establish the diagnosis of PBC in most cases [16], UDCA is the first-line therapy for PBC [17] and interventional therapy but not UDCA is the main treatment for most patients with secondary biliary cholangitis [18], therefore the epidemiological characteristics of PBC in Taiwan might be comprehensively examined by using the ICD-9-CM code, biochemical parameters and treatment data to define patients with PBC in the TNHIRD, which provides the medical information of a nationwide population of 26,573,661 persons, including the National Health Insurance (NHI) administrative database, the Cancer Registry Database, and the Death Registry Database. The mandatory, single-payer NHI program provides comprehensive coverage including ambulatory care, hospital services, laboratory tests, and prescription drugs. More than 99% of the population is enrolled in the program, and approximately 92% of healthcare organizations are contracted with the NHI Administration. However, the use of the ICD-9-CM code is not a prerequisite for the prescription of UDCA for PBC in Taiwan. Some patients might have never been assigned the ICD-9-CM code, but were diagnosed with PBC based on their results for Alk-p and AMA, then treated with UDCA if it was not contraindicated. These patients could also have complete records of their biochemical results and medications in the TNHIRD despite the fact that they were never assigned the ICD-9-CM code. Thus, four PBC cohorts were defined as follows:

PBC cohort 1 (C1): ICD-9-CM code (571.6)

PBC cohort 2 (C2): Alk-p and AMA measurements

PBC cohort 3 (C3): Alk-p and AMA measurements and UDCA treatment

PBC cohort 4 (C4): ICD-9-CM code (571.6), Alk-p and AMA measurements, and UDCA treatment

For these PBC cohorts, the date a patient was first assigned the ICD-9-CM code or underwent the first AMA test was assumed to be the index date of diagnosis, and the data from this date were considered the baseline data. Only those patients who met the definitions for C1, C2, C3, or C4 for the first time when they were ≥18 years old were enrolled. The outcomes, including liver cirrhosis, hepatocellular carcinoma (HCC), liver transplantation (LT), dyslipidemia, osteoporosis, and mortality, were recorded. Information on HCC diagnosis was retrieved from the Cancer Registry Database. Subjects were observeduntil the date of death or the end of follow-up (31 December 2015), whichever came first.

### 2.2. Statistics

All statistical analyses were performed using the Statistical Analysis Software (SAS version 9.4, SAS Institute Inc., Cary, NC, USA). Continuous variables are summarized as the mean ± standard deviations, and categorical variables are summarized as numbers and percentages (%). To compare variables in different groups, continuous variables were analyzed using Student’s t-tests, whereas categorical variables were analyzed using chi-squared or Fisher’s exact tests, as appropriate. Data were analyzed by one-way analysis of variance (ANOVA) when comparing variables among multiple groups. The annual national prevalence rate of PBC was determined as the number of PBC cases divided by the number of adult NHI enrollees in the corresponding year from 2002 to 2015. We used a 4-year washout period (2002–2005) [15] to calculate the annual incidence rate of PBC from 2006 to 2015 while excluding prevalent cases due to the data recording beginning in 2002. The annual incidence rates of PBC were calculated as the number of new cases of PBC per 100,000 people per year. Age- and sex-adjusted rates were calculated by dividing the expected number of cases in each age and sex group by the age- and sex-specific standard population. For the standard population, we used the population of inhabitants at the end of 2010 from the Department of Household Registration Affairs, Ministry of the Interior of Taiwan, as the NHI covers >99% of the population in Taiwan. The average annual percentage change, analyzed by the JoinPoint regression program (Version 4.7.0.0), was adopted to evaluate trends in the annual prevalence and incidence rates. Data were analyzed by ANOVA when comparing variables among multiple groups. Post hoc analyses were performed with the least significant difference multiple comparison test. Statistical significance was defined at the 5% level.

### 2.3. Primary and Secondary Outcomes

The primary outcomes were the prevalence and incidence rates in the most representative PBC cohort in Taiwan; the secondary outcomes were the hepatic and extrahepatic complications in that representative cohort.

### 2.4. Institutional Review Board

The study was conducted in accordance with good clinical practice and all applicable regulations, including the Declaration of Helsinki and local regulatory requirements, and was approved by the institutional ethics committee. The need to obtain consent was waived due to the national data used in this study being deidentified by encrypting any personally identifying information.

## 3. Results

### 3.1. Baseline Characteristics

**Figure 1:**
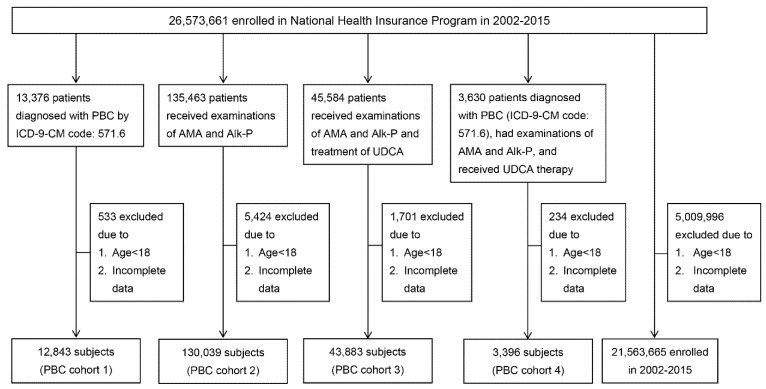
A schematic flowchart of patient enrollment. PBC: primary biliary cholangitis;ICD-9-CM: International Classification of Disease, Ninth Revision, Clinical Modification; AMA: antimitochondrial antibodies; Alk-P: alkaline phosphatase; UDCA: ursodeoxycholic acid; HBV: hepatitis B virus; HCV: hepatitis C virus; YO: years old.

As shown in Figure 1 and Table 1, 12,843 (females: 6235 (48.55%)), 130,093 (females: 74,191(57.03%)), 43,883 (females: 23,959 (54.60%)) and 3396 (females: 2533 (74.59%)) individuals met the criteria for C1, C2, C3 and C4, respectively. The mean age ranged from 53.73 years (C2) to 57.61 years (C4). The female-to-male ratio ranged from 0.943 (C1) to 2.93 (C4). Among the four cohorts, C4 had the highest female-to-male ratio and mean age, and C2 had the lowest mean age.

### 3.2. Prevalence Rates

**Figure 2:**
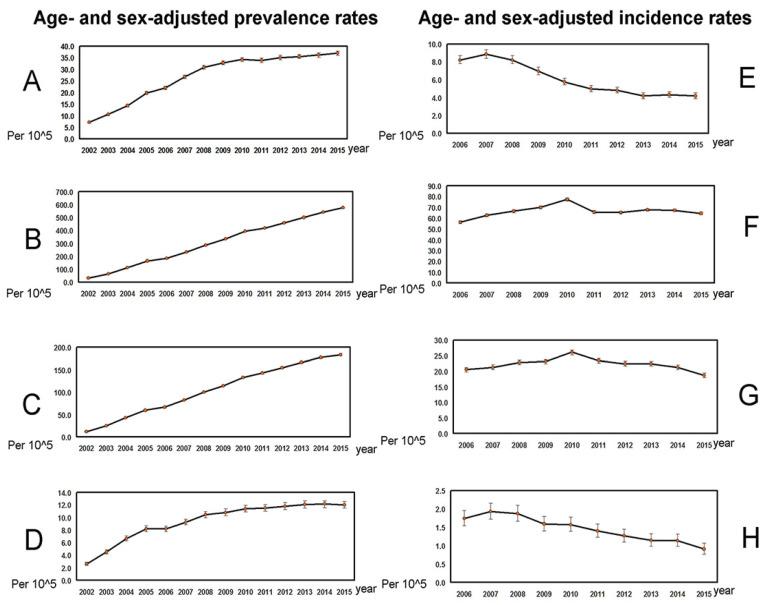
The trends in age- and sex-adjusted prevalence (**A**–**D**) and incidence (**E**–**H**) rates (per 10^5^; mean ± 95% confidence interval of prevalence rates). (**A**), Prevalence rates in PBC cohort 1. (**B**), Prevalence rates in PBC cohort 2. (**C**), Prevalence rates in PBC cohort 3. (**D**), Prevalence rates in PBC cohort 4. (**E**), Incidence rates in PBC cohort 1. (**F**), Incidence rates in PBC cohort 2. (**G**), Incidence rates in PBC cohort 3. (**H**), Incidence rates in PBC cohort 4.

As shown in Table 2, in the 21,536,665 subjects (Figure 1) enrolled from 2002 to 2015, the average annual age- and sex-adjusted prevalence rates ranged from 9.419/10^5^ (C4) to 307.658/10^5^ (C2). The average age-adjusted prevalence rate of PBC ranged from 14.74/10^5^ (C4) to 366.428/10^5^ (C2) for females and from 4.028/10^5^ (C4) to 248.106/10^5^ (C2) for males, with the female-to-male ratio ranging from 1.192 (C1) to 3.66 (C4). C4 had the lowest prevalence rate and highest female-to-male ratio among the four cohorts. There were increasing trends in the age- and sex-adjusted prevalence rates for all four cohorts (Figure 2A–D, Table 2).

### 3.3. Incidence Rates

As shown in Table 3, from 2006 to 2015, the average annual age- and sex-adjusted incidence rates ranged from 1.456/10^5^ (C4) to 66.386/10^5^ (C2), the average age-adjusted incidence rates ranged from 2.093/10^5^ (C4) to 74.045/10^5^ (C2) for females and from 0.810/10^5^ (C4) to 58.634/10^5^ (C2) for males. The female-to-male ratio ranged from 0.850 (C1) to 2.583 (C4). C4 had the lowest incidence rate and highest female-to-male ratio among the four cohorts. There were decreasing trends in the annual incidence rates in C1 (−9.09%, *p* < 0.0001) and C4 (−6.68%, *p* < 0.0001), while the incidence rates remained steady in C2 and C3 (Figure 2E–H, Table 3).

### 3.4. Hepatic and Extrahepatic Outcomes

The prevalence rates of incident outcomes in PBC patients are listed in Table 4. The cirrhosis rates ranged from 7.21% (C2) to 39.34% (C4), the HCC rates ranged from 2.77% (C2) to 6.66% (C1), and the LT rates ranged from 1.07% (C2) to 6.77% (C4). The osteoporosis rates ranged from 7.06% (C2) to 13.87% (C4), and the dyslipidemia rates ranged from 10.82% (C1) to 17.21% (C4). The mortality rates ranged from 18.24% (C2) to 47.36% (C1). Among the four cohorts, C4 had the highest rates of cirrhosis, LT, osteoporosis and dyslipidemia; C1 had the highest rates of HCC and mortality; and C2 had the lowest rates of cirrhosis, HCC, LT, osteoporosis and mortality.

## 4. Discussion

Consistent with most studies [13,19], the prevalence rates increased over time in all PBC cohorts, reflecting the fact that most patients diagnosed with PBC, especially those treated with UDCA, have an expected lifespan that does not differ from that of the general population [1]. The inclusion criteria for the various PBC cohorts accounted for the differences in the numbers of enrolled patients: C2 and C4 had the highest and lowest numbers (and prevalence and incidence rates), respectively, as they had the least and most strict definitions of PBC, respectively. The prevalence rates of PBC thus ranged from 9.419/10^5^ (C4) to 307.658/10^5^ (C2), and the incidence rates ranged from 1.456/10^5^ (C4) to 66.386/10^5^ (C2) in Taiwan. Given that the range of prevalence rates for PBC was 1.91–40.2/10^5^ [20,21], and the range of incidence rates was 0.33–5.8/10^5^ [20], only C1 (prevalence rate: 25.8/10^5^; incidence rate: 6.062/10^5^) and C4 were within the reported ranges; thus, C1 and C4 might be the most accurately representative PBC cohorts in Taiwan based on the prevalence and incidence rates. With regard to the female-to-male ratio, which was 1.192, 1.477, 1.30 and 3.66 in C1, C2, C3 and C4, respectively, the ratio of 3.66 in C4 is the most accurate representation of the degree of female predominance in the population of patients with PBC in Taiwan. The female-to-male ratio was found to be 4:1 in Hong Kong [10], which is also located in Asia and has a population of the same ethnicity as that of Taiwan. The female-to-male ratio in the PBC cohort in Hong Kong was based on the definition of PBC according to the ICD-9-CM code [10]. Although the median female-to-male ratio in the population with PBC is generally accepted to be 10:1 [4], a declining trend in the female-to-male ratio in patients with PBC had been reported recently [15], and it has been suggested that PBC may have been under-diagnosed in men in early studies.

Notably, most PBC studies have shown that the incidence rates either remained steady [13,19] or increased over time [22]. In contrast, although C4 had reasonable prevalence and incidence rates [20,21] and a similarly reasonable female-to-male ratio [10,15], it showed a clear decreasing trend in the incidence rate. As the risk of PBC has increased in areas with high levels of socioeconomic deprivation [23], the decrease in the incidence rate might reflect the profound socioeconomic improvement in the Taiwanese population. On the other hand, it might also indicate that the criteria used to establish the diagnosis of PBC in this cohort were too strict to enroll new cases. Future prospective studies based on comprehensive ICD-10-CM codes to survey the precise trends in the annual incidence rates of PBC in Taiwan are needed.

The fact that C2 had the lowest rates of cirrhosis, HCC, and LT among the four cohorts suggests that C2 was composed of patients with early liver disease rather than PBC; thus, the prognosis was the best in that cohort even though no UDCA was prescribed. The fact that C2 had the lowest mean age also supports the findings of the least severe outcomes in this cohort. Interestingly, C4, the candidate cohort that most accurately represented Taiwanese PBC patients, yielded the highest rate of cirrhosis, 39.34%, which reflects the fact that only 60–70% of patients with PBC treated with UDCA exhibit complete responses [5]. C4 also had the highest mean age of the four cohorts. However, the highest rates of HCC and mortality were noted in C1, rather than in C4. This phenomenon indicates that a substantial proportion of patients with secondary biliary cirrhosis might have been included into C1, leading to the relatively worse prognosis in C1 than in any of the other cohorts. This might partially account for the higher LT rate in C4 than in C1, as LT is not recommended for most cases of secondary biliary cirrhosis, while up to 40% of PBC patients with suboptimal responses to UDCA require LT, or second-line treatment [24] with OCA [6] which is not yet available in Taiwan. These differences in outcomes between C1 and C4 suggest that defining PBC with biochemistry measurements and UDCA treatment, in addition to the ICD-9-CM code, increases the representativeness of C4 for PBC patients in Taiwan. Consistent with this finding, C4 had the highest rates of osteoporosis and dyslipidemia, both of which are common extrahepatic complications of PBC [7]. Moreover, the mortality of C4 (34.22%) was within the reported range of mortality in PBC patients [25] and supported the reliability of C4 as the representative PBC corhort in Taiwan. As mentioned above, some unregistered (i.e., lacking ICD-9-CM (571.6) data) patients might be diagnosed with PBC based on biochemistry data, and treated with UDCA. Those patients were categorized into C3. Although C3 was not the most representative PBC cohort for Taiwan in all aspects, the inclusion of some unregistered PBC patients in C3 might account for the underestimations of the prevalence and incidence rates in C4. Thus, C3 provides the extreme upper limits of the prevalence (104.254/10^5^) and incidence (22.217/10^5^) rates of PBC in Taiwan.

There are limitations of the current study. First, as previously mentioned, the ICD-9-CM code 571.6 includes cases of secondary biliary cirrhosis, which might bias the data in C1 and C4, although we attempted to reduce this bias by adding the Alk-p and AMA measurements and UDCA treatment as enrollment criteria for C4. Given that the TNHIRD does not contain direct laboratory results, and UDCA treatment is not specific to PBC, even subjects who met the criteria for C4 are not necessarily true cases of PBC. Second, hepatitis B virus (HBV) infection is hyperendemic, and hepatitis C virus (HCV) infection is wide spread in Taiwan [26]. Both HBV [27] and HCV infections [28,29] are associated with many hepatic and extrahepatic complications, which might bias the outcomes in all four PBC cohorts. Third, some primary sclerosing cholangitis (PSC) patients with cirrhosis might have been categorized into C4, while the portion of PSC in C4 should be negligible, since PSC is very rare in Taiwan [30]. Fourth, PBC-specific antinuclear antibodies (ANAs), including anti-sp100 and anti-gp210, might aid to diagnose AMA-negative PBC [4]. However, these autoantibodies were not assessed in most labs in Taiwan, and the associated data thus are not available in TNHIRD. A future prospective study of PBC patients who were all registered with the ICD-10-CM code K74.3,with comprehensive surveys of anti-sp100 and anti-gp210, and are not infected with HBV or HCV, is needed to precisely verify the prevalence, incidence and outcomes of PBC in Taiwan.

**Figure 3:**
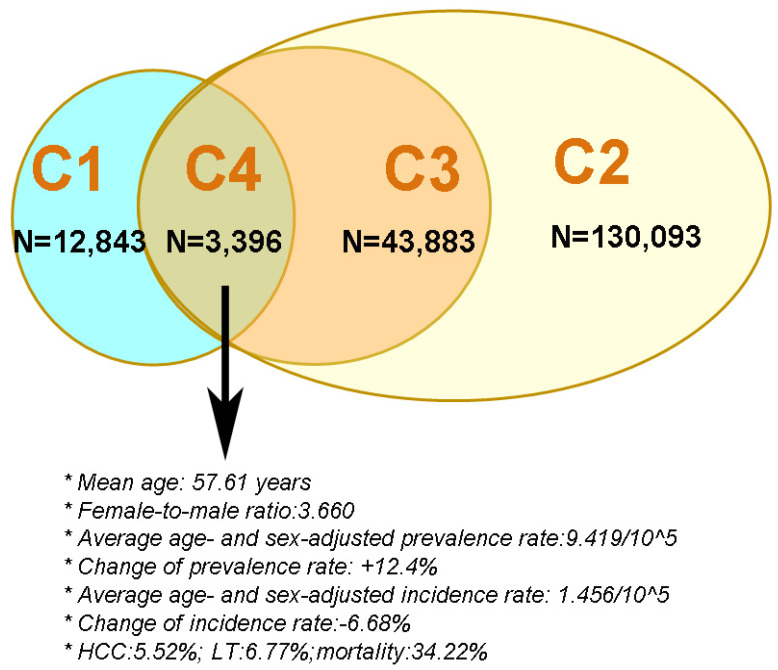
The criteria for and sample size of the 4 primary biliary cholangitis cohorts in Taiwan. C: cohort; blue circle: PBC cohort 1; green oval; PBC cohort 4; brown circle: PBC cohort 3; yellow oval: PBC cohort 2; HCC: hepatocellular carcinoma; LT: liver transplantation.

A summary of the characteristics of the 4 cohorts, particularly C4, is shown in Figure 3.

Taken together, the cohort that most accurately represented PBC patients in Taiwan was C4, with average age- and sex-adjusted prevalence and incidence rates of 9.419/10^5^ and 1.456/10^5^, respectively. Both rates were within the ranges reported worldwide, and the female-to-male ratio was 3.66, which is close to what was reported in other Asian countries. The prevalence rates increased over time, while the incidence rates decreased. In this PBC cohort, 39.34% had cirrhosis, 5.52% had HCC, 6.77% underwent LT, and 34.22% died within 14 years.

## Figures and Tables

**Table 1:** Baseline characteristics of the 4 PBC cohorts.

	PBC Cohort 1	PBC Cohort 2	PBC Cohort 3	PBC Cohort 4	ANOVA or Chi-sqTest *p* Values	Post-Hoc: *t*-Test or Chi-sqTest *p* Values
Criteria	ICD-9-CM: 571.6	AMA+AlK-P	AMA+AlK-P+ UDCA	ICD-9-CM:571.6+ AMA+AlK-P+UDCA		
Total (N)	12,843	130,093	43,883	3396		
Female (N)	6235	74,191	23,959	2533		
Male (N)	6608	55,902	19,924	863		
Female-to-male ratio	0.943	1.32	1.2	2.93	<0.0001	C1 vs. C2: <0.0001 C1 vs. C3: <0.0001 C1 vs. C4: <0.0001 C2 vs. C3: <0.0001 C2 vs. C4: <0.0001 C3 vs. C4: <0.0001
Age (years), mean +/− SD (range) years	57.27 ± 14.88 (18–103)	53.73 ± 16.00 (18–105)	55.60 ± 14.90 (18–102)	57.61 ± 13.79 (18–98)	0.00125	C1 vs. C2: <0.0001 C1 vs. C3: <0.0001 C1 vs. C4: <0.0001 C2 vs. C3: <0.0001 C2 vs. C4: <0.0001 C3 vs. C4: <0.0001

PBC: primary biliary cholangitis; ICD-9-CM: International Classification of Disease, Ninth. Revision, Clinical Modification; AMA: antimitochondrial antibody; AlK-P: alkaline phosphatase; UDCA: ursodeoxycholic acid; N: total enrolled cases (2002–2015); SD: standard deviation; C1: PBC cohort 1; C2: PBC cohort 2; C3: PBC cohort 3; C4: PBC cohort 4.

**Table 2:** Prevalence rates of the 4 PBC cohorts.

	PBC Cohort 1	PBC Cohort 2	PBC Cohort 3	PBC Cohort 4	ANOVA *p* Values	Post-Hoc: *t*-Test *p* Values
Average overall age- and sex-adjusted prevalence rate	26.832/10^5^	307.658/10^5^	104.254/10^5^	9.419/10^5^	<0.0001	C1 vs. C2: <0.0001 C1 vs. C3: 0.2116 C1 vs. C4: 1.0000 C2 vs. C3: <0.0001 C2 vs. C4: <0.0001 C3 vs. C4: 0.0642
Change inprevalence rates (% and *p* values ^a^)	13.38 (<0.0001)	24.92 (<0.0001)	23.37 (<0.0001)	12.40 (<0.0001)		
Average female age-adjusted prevalence rate	29.168/10^5^	366.428/10^5^	117.887/10^5^	14.740/10^5^	<0.0001	C1 vs. C2: <0.0001 C1 vs. C3: 0.1950 C1 vs. C4: 1.0000 C2 vs. C3: <0.0001 C2 vs. C4: <0.0001 C3 vs. C4: 0.0817
Average male age-adjusted prevalence rate	24.464/10^5^	248.106/10^5^	90.439/10^5^	4.028/10^5^	<0.0001	C1 vs. C2: <0.0001 C1 vs. C3: 0.2387 C1 vs. C4: 1.0000 C2 vs. C3: <0.0001 C2 vs. C4: <0.0001 C3 vs. C4: 0.0475
Female-to-male ratio	1.192	1.477	1.303	3.660	<0.0001	C1 vs. C2: 0.7037 C1 vs. C3: 1.0000 C1 vs. C4: <0.0001 C2 vs. C3: 1.0000 C2 vs. C4: <0.0001 C3 vs. C4 <0.0001

PBC: primary biliary cholangitis; C1: PBC cohort 1; C2: PBC cohort 2; C3: PBC cohort 3; C4: PBC cohort 4; ^a^ Test for trend of annual prevalence rate in each cohort.

**Table 3:** Incidence rates of the 4 PBC cohorts.

	PBC Cohort 1	PBC Cohort 2	PBC Cohort 3	PBC Cohort 4	ANOVA *p* Values	Post-Hoc: *t*-Test *p* Values
Average overall age- and sex-adjusted incidence rate	6.062/10^5^	66.386/10^5^	22.217/10^5^	1.456/10^5^	<0.0001	C1 vs. C2: <0.0001 C1 vs. C3: <0.0001 C1 vs. C4: 0.0102 C2 vs. C3: <0.0001 C2 vs. C4: <0.0001 C3 vs. C4: <0.0001
Change inincidence rates (% and *p* values ^a^)	−9.09 (<0.0001)	1.42 (0.3424)	−0.49 (0.5860)	−6.68 (<0.0001)		
Average female age-adjusted incidence rate	5.576/10^5^	74.045/10^5^	23.751/10^5^	2.093/10^5^	<0.0001	C1 vs. C2: <0.0001 C1 vs. C3: <0.0001 C1 vs. C4: 0.2633 C2 vs. C3: <0.0001 C2 vs. C4: <0.0001 C3 vs. C4: <0.0001
Average male age-adjusted incidence rate	6.554/10^5^	58.634/10^5^	20.663/10^5^	0.810/10^5^	<0.0001	C1 vs. C2: <0.0001 C1 vs. C3: <0.0001 C1 vs. C4: 0.0092 C2 vs. C3: <0.0001 C2 vs. C4: <0.0001 C3 vs. C4: <0.0001
Female-to-male ratio	0.850	1.262	1.149	2.583	<0.0001	C1 vs. C2: 0.0214 C1 vs. C3: 0.2160 C1 vs. C4: <0.0001 C2 vs. C3: 1.0000 C2 vs. C4: <0.0001 C3 vs. C4: <0.0001

PBC: primary biliary cholangitis; C1: PBC cohort 1; C2: PBC cohort 2; C3: PBC cohort 3; C4: PBC cohort 4; ^a^ Test for trend of annual prevalence rate in each cohort.

**Table 4:** Prevalence rates of various complications of 4 PBC cohorts.

	Complications	PBC Cohort 1	PBC Cohort 2	PBC Cohort 3	PBC Cohort 4	Chi-sqTest *p* Values	Post-Hoc: Chi-sqTest *p* Values
**Hepatic**							
	LC	17.86%	7.21%	14.29%	39.34%	<0.0001	C1 vs. C2: <0.0001 C1 vs. C3: <0.0001 C1 vs. C4: <0.0001 C2 vs. C3: <0.0001 C2 vs. C4: <0.0001 C3 vs. C4: <0.0001
	HCC	6.66%	2.77%	5.21%	5.52%	<0.0001	C1 vs. C2: <0.0001 C1 vs. C3: <0.0001 C1 vs. C4: 0.1028 C2 vs. C3: <0.0001 C2 vs. C4: <0.0001 C3 vs. C4: 1.0000
	LT	2.80%	1.07%	2.52%	6.77%	<0.0001	C1 vs. C2: <0.0001 C1 vs. C3: 0.5210 C1 vs. C4: <0.0001 C2 vs. C3: <0.0001 C2 vs. C4: <0.0001 C3 vs. C4: <0.0001
**Extrahepatic**							
	Osteoporosis	7.44%	7.06%	7.77%	13.87%	<0.0001	C1 vs. C2: 0.8371 C1 vs. C3: 1.0000 C1 vs. C4: <0.0001 C2 vs. C3: <0.0001 C2 vs. C4: <0.0001 C3 vs. C4: <0.0001
	Dyslipidemia	10.82%	14.10%	14.80%	17.21%	<0.0001	C1 vs. C2: <0.0001 C1 vs. C3: <0.0001 C1 vs. C4: <0.0001 C2 vs. C3: 0.0105 C2 vs. C4: <0.0001 C3 vs. C4: 0.0059
**Mortality**		47.36%	18.24%	23.20%	34.22%	<0.0001	C1 vs. C2: <0.0001 C1 vs. C3: <0.0001 C1 vs. C4: <0.0001 C2 vs. C3: <0.0001 C2 vs. C4: <0.0001 C3 vs. C4: <0.0001

PBC: primary biliary cholangitis; LC: liver cirrhosis; HCC: hepatocellular carcinoma; LT: liver transplantation; C1: PBC cohort 1; C2: PBC cohort 2; C3: PBC cohort 3; C4: PBC cohort 4.

## Data Availability

The datasets generated during and/or analysed during the current study are available from the corresponding author on reasonable request.
